# Limit Equilibrium Method-based Shear Strength Prediction for Corroded Reinforced Concrete Beam with Inclined Bars

**DOI:** 10.3390/ma12071014

**Published:** 2019-03-27

**Authors:** Yafei Ma, Baoyong Lu, Zhongzhao Guo, Lei Wang, Hailong Chen, Jianren Zhang

**Affiliations:** 1 School of Civil Engineering, Changsha University of Science & Technology, Changsha 410114, China; lubyong@163.com (B.L.); guozzhao@163.com (Z.G.); 2 Key Laboratory of Bridge Engineering Safety Control by Department of Education, Changsha 410114, China; 3 Department of Mechanical Engineering, University of Kentucky, Lexington 40506, KY, USA; hailong.chen@uky.edu

**Keywords:** reinforced concrete, shear strength, corrosion, inclined bar, diagonal crack

## Abstract

Shear strength is a widely investigated parameter for reinforced concrete structures. The corrosion of reinforcement results in shear strength reduction. Corrosion has become one of the main deterioration factors in reinforced concrete beam. This paper proposes a shear strength model for beams with inclined bars based on a limit equilibrium method. The proposed model can be applied to both corroded and uncorroded reinforced concrete beams. Besides the tensile strength of longitudinal steel bars, the shear capacity provided by the concrete on the top of the diagonal crack, the tensile force of the shear steel at the diagonal crack, the degradation of the cross-sectional area and strength of the reinforcements induced by corrosion are all considered. An experimental work on two groups accelerated corroded beams was performed. Good agreements were found between the proposed theoretical predictions and experimental observations.

## 1. Introduction

The corrosion of reinforcement is an important factor contributing to the performance deterioration of existing reinforced concrete (RC) structures [1,2,3,4]. Corrosion in natural environment is an electrochemical process, which is mainly induced by chloride ions. Corrosion initiates when the harmful ions diffuse to the reinforcement surface and reach a threshold concentration [5]. Corrosion decreases the yield strength and elongation of steel bars [6]. With the continuous growth of corrosion level, an increased volume of corrosion product causes concrete cracking [7,8], which leads to a bond loss between concrete and reinforcement and ultimately reduces the service life of structures [9,10,11]. Over the past decades, several researchers have studied the flexural properties of concrete members with corroded longitudinal steel [12,13]. However, few works focus on the shear behavior of aging RC members. Engineering investigations have found that corrosion often occurs earlier in stirrups than that in other types of reinforcements since stirrups are located at the most periphery of the steel skeleton [14,15,16]. The stirrups generally have a larger corrosion degree value compared with longitudinal or inclined rebars. The collapse form for RC members may change from the flexural failure of normal section to the shear collapse in oblique section as corrosion propagates [17,18]. In addition, the deterioration due to corrosion of shear reinforcing bars may cause an embrittlement and sudden failure mode of beams.

The deterioration of shear behavior induced by corrosion is very complex and depends on many factors. Previous experimental investigations [19,20] show that the shear capacity reduces when the stirrup corrosion exceeds a certain level. Juarez et al. [21] indicated that the bond degradation between concrete and stirrups affects the restraining ability of shear cracks. They found that the maximum shear strength for severely corroded members was reduced by nearly 30%. El-Sayed [22] suggested a set of design methods to evaluate the remaining shear capacity of stirrup-deteriorated RC members. The observed corrosion rust-caused crack size is applied to represent the corrosion degree in this method. The shear strength is also influenced by longitudinal reinforcement deterioration, reduction of the effective section of beam, and corrosion cracks in compressed zone of concrete beam [23,24,25]. To identify the prominent influential parameters, previous work in the corroded longitudinal rebars related to the members without or with undamaged stirrups [26], which may differ from the real conditions. In practical situation, the corrosion of stirrups and longitudinal rebars often occurs simultaneously. Xia et al. [27] examined the shear behavior of concrete members that have various damage degrees on longitudinal rebars and stirrups. They found that the mean crack width is most closely related to the decrease of shear strength caused by corrosion.

According to experimental observations, several analysis models have been established to investigate the shear capacity of RC members. These models consists of theoretical and empirical types [28]. Vecchio and Collins [29] suggested a shear design procedure using an improved compressed field method. Results based on this method reveal that the current procedure may underestimate real capacity of structural components. Azam et al. [30] found that corrosion may change the load transfer form. A strut and tie model is also proposed to assess the shear capacity of deteriorated concrete members. Ghahremannejad and Abolmaali [31] developed a new ultimate shear strength model for performing displacement controlled analysis of distributed loads, but the effect of steel bar corrosion was ignored. The empirical-based models require a reliable database of test data. Based on existing experimental results, Ahmad and Bhargava [32] reported shear strength data for concrete beams that do not have transverse steel bars. Lu et al. [33] performed a comparison study on nine existing theoretical shear capacity models, and then developed a new empirical shear strength model combining the observed data from literature. Wang et al. [34] proposed a modified shear strength prediction model based on ACI code. In this model, the area loss of deteriorated shear steel bars and concrete damage are included. These predictive models are derived based on mechanics, while the coefficients are obtained based on fitting experimental results. For further calibration, many experimental data with a wide range corrosion levels for different types of rebars are also required.

In addition, most of the above studies investigate the corrosion-induced shear behavior deterioration in RC beams with stirrups perpendicular to the longitudinal steel bar. Some design codes [35] indicate that the shear reinforcements are allowed to have inclined bars with an angle 45° with the longitudinal reinforcement. The combined action of stirrups and diagonal reinforcement is a more effective way to improve the shear resistance of RC structures and is widely used in early-built bridges. Corrosion leads to a stress redistribution in RC members, which may affect the contribution of inclined bars to shear resistance. Zhang et al. [36] suggested a theoretical shear strength model of RC members with inclined rebar. However, they did not consider the corrosion of steel bars, and only used to two beams for validation.

Based on above discussion, the current study aimed to investigate the influences of mass loss induced by corrosion on the shear strength degradation of RC members subjected to different corrosion conditions. This study is paper as follows. First, a shear strength model with inclined bars is formulated based on a limit equilibrium method. The shear forces provided by the corroded rebars and non-cracked concrete are discussed. Next, the test design and experimental results are presented. Following this, the proposed model is verified using experimental observations. Finally, some conclusions are drawn and future suggestions are made based on the present study.

## 2. Shear Strength Model for Corroded Reinforced Concrete Members

**Figure 1:**
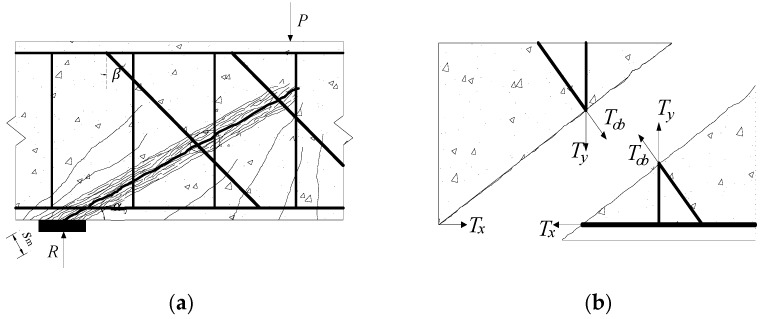
Shear failure and diagonal crack reinforcement internal force: (**a**) shear compression failure; and (**b**) schematic diagram of steel bars at the diagonal crack.

The constraint provided by stirrups onto concrete decreases as corrosion level increases, accelerating the propagation of the diagonal cracks. This may weaken the bite force between the aggregates on both sides of the diagonal crack [37]. Previous studies indicate that the shear stress effect of inclined steel bars and stirrups has an insignificant effect on the shear strength [12]. Therefore, the shear strength model of RC beam proposed in this study is composed of the tensile strength of longitudinal steel bars, the shear force provided by the concrete on the top of the diagonal crack and the tensile force of the shear steel at the diagonal crack. Besides, the degradation in the cross-sectional area and strength of reinforcement induced by corrosion are also considered. Figure 1 shows the schematic of a failed beam and the critical crack.

### 2.1. Capacity of Corroded Rebars

Corrosion reduces the area and yield strength of rebars. The yield strength of rebars reduces approximately linearly as corrosion level increases, which can be expressed as [13,38]

(1) $$f_{y}=(1.0-\alpha_{y}\eta_{s}\times 100)\cdot f_{y0}$$

where $f_{y0}$ and $f_{y}$ are yield strength of uncorroded and corroded rebars, respectively; $\eta_{s}$ is corrosion level; and $\alpha_{y}$ is an empirical coefficient which related to the material properties in specific adverse environments and is generally obtained from experiments. Here, $\alpha_{y}$ is set to 0.009 [13].

The remaining area of corroded rebar is written as

(2) $$A_{\mathrm{eff}}=\frac{\pi D_{0}{}^{2}}{4}(1-\eta_{s})$$

where *A*_eff_ is effective area and *D*_0_ is the diameter of uncorroded rebar.

The tensile forces of various types of steel bars (as shown in Figure 1) can be determined as

(3) $$T_{x}=A_{\mathrm{eff},x}f_{\mathrm{yc},x}$$

(4) $$T_{y}=A_{\mathrm{eff},y}f_{\mathrm{yc},y}$$

(5) $$T_{\mathrm{ob}}=A_{\mathrm{eff},\mathrm{ob}}f_{\mathrm{yc},\mathrm{ob}}$$

where *T_x_* and *T*_y_; and *T*_ob_, *f*_yc,x_, and *f*_yc,y_; and *f*_yc,ob_, *A*_eff,x_, *A*_eff,y_ and *A*_eff,ob_ are the tensile forces, yield strengths and effective areas of the longitudinal reinforcements, stirrups, and diagonal steel bars, respectively.

### 2.2. Capacity of Compression Concrete

**Figure 2:**
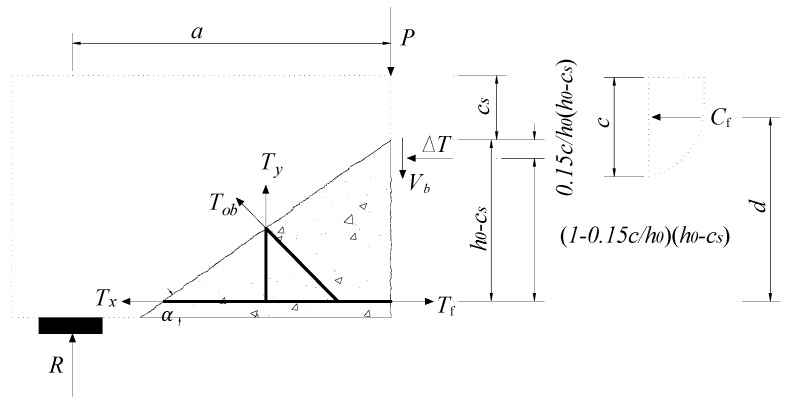
Triangle element isolation body force diagram.

The tip of critical diagonal crack is generally located above that of the flexural cracks below the loading point when shear failure occurs. Figure 2 shows the forces on triangular concrete element below critical diagonal crack. ∆*T* represents the resultant force of concrete compressive stress in *c*–*c*_s_ region. The distance from the resultant force to the tip of critical diagonal crack is approximately 0.15(*c*/*h*_0_)(*h*_0_−*c*_s_) [18]. According to the equilibrium of moments acting on ∆*T*, the following expressions can be obtained

(6) $$0.5(T_{y}+T_{\mathrm{ob}}\cos\beta )\frac{h_{0}-c_{s}}{\tan\alpha }+T_{\mathrm{ob}}\sin\beta (h_{0}-c_{s})(0.5-0.15\frac{c}{h_{0}})+(T_{x}-T_{f})(1-0.15\frac{c}{h_{0}})(h_{0}-c_{s})=0$$

where *h*_0_ is the effective height of beam; $c_{s}$ is the depth of compression zone; *c* is the height of the compression zone; α is an angle between critical diagonal crack and main reinforcement; and β is an angle between the diagonal steel bar and the stirrup.

Taking the rectangular concrete element in the dashed line as the isolator, the moment balance acting on the resultant of concrete stress point *C*_f_ gives

(7) $$T_{f}=Ra/d$$

where *T*_f_ is the longitudinal steel force in pure bending region; *R* is the beam capacity, which is equal to the external shear force *P*; a represents horizontal length from loading point to support; and *d* represents vertical length from longitudinal reinforcement to point *C*_f_.

**Figure 3:**
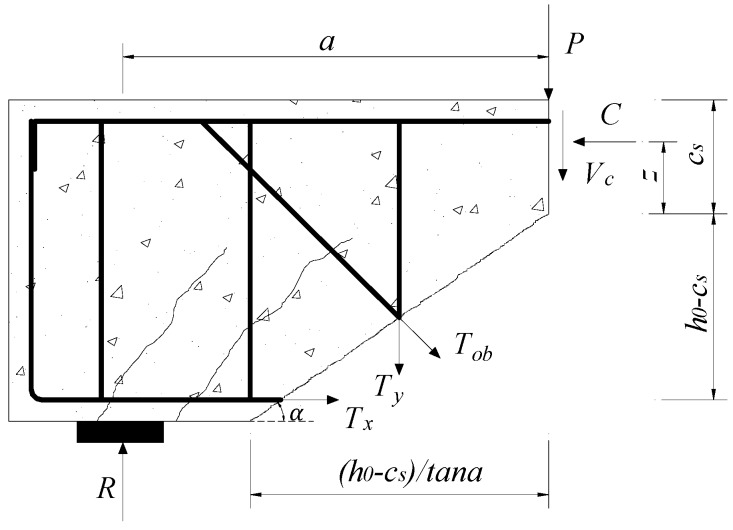
Forces on free-body diagram above critical diagonal crack.

The height of *c*_s_ above the critical diagonal crack is a main influential factor for shear strength. Figure 3 shows the forces acting on a free-body diagram above the critical diagonal crack. A moment equilibrium condition at the resultant force point *C* of concrete compressive zone can be calculated as

(8) $$Ra-(T_{y}+T_{\mathrm{ob}}\cos\beta )[0.5(h_{0}-c_{s})/\tan\alpha ]-T_{\mathrm{ob}}\sin\beta [0.5(h_{0}-c_{s})+z]-T_{x}(h_{0}-c_{s}+z)=0$$

where *z* represents a length from the tip of critical diagonal crack to resultant force of concrete compression zone.

The distance from the extreme compression fiber of beam to the resultant force point of concrete compression zone is approximately 0.4*c* [18], which yields $c/h_{0}\approx 0.5$, $\tan\alpha \approx h_{0}/a$, and $d/h_{0}\approx 1-0.4c/h_{0}$. Combining Equations (6)–(8), the above expressions can be rewritten as

(9) $$h_{0}-c_{s}=\frac{T_{x}(0.8h_{0}-z)+0.43T_{y}a+T_{\mathrm{ob}}\;(0.43a\cos\beta +0.37h_{0}\sin\beta -z\sin\beta )}{0.5T_{y}a/h_{0}+0.5T_{\mathrm{ob}}(a/h_{0}\cos\beta +\sin\beta )+T_{x}}$$

**Figure 4:**
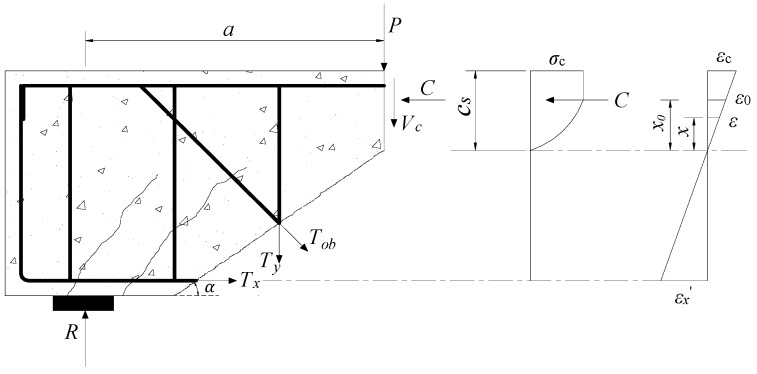
Calculating model diagram.

Figure 4 shows the schematic diagram of the shear strength model. The stress is assumed to be linearly distributed along the height of the shear-compression zone. According to the assumption of plane section, the horizontal concrete strain *ε* at any point within *c*_s_ range of the height of the shear-compression zone of critical diagonal crack is calculated by

(10) $$\varepsilon =\frac{\varepsilon_{0}x}{x_{0}}=\frac{\varepsilon_{c}x}{c_{s}}=\frac{\varepsilon_{x}^{\prime }x}{h_{0}-c_{s}}$$

where $\varepsilon_{0}$ is strain under the peak stress of concrete and has the value of 0.002 [39]; $\varepsilon_{c}$ is the compressive edge strain of concrete; $\varepsilon_{x}^{\prime }$ is the longitudinal steel strain; *x* represents the distance between *ε* position and neutral axis; and *x*_0_ represents the height corresponding to location of the peak stress of the concrete.

The stress–strain constitutive relationship of concrete is determined as [18]

(11) $$\sigma (\varepsilon )=\left\{ \begin{array}{cc} f_{c}[1-{\left(1-\frac{\varepsilon_{c}}{\varepsilon_{0}}\right)}^{n}], & \varepsilon \leq \varepsilon_{0}\\ f_{c}, & \varepsilon_{0}<\varepsilon \leq \varepsilon_{\mathrm{cu}} \end{array}\right.$$

where *n* is a coefficient, with $n=2-\left(f_{\mathrm{cu},k}-50\right)/60$ for *n* ≤ 2, and otherwise *n* = 2; *f*_c_ represents designed concrete axial compressive strength; *f*_cu,k_ represents standard value of cubic compressive strength of concrete; and $\varepsilon_{\mathrm{cu}}$ represents ultimate compressive strain of normal section.

Based on Equations (10) and (11), the resultant force in concrete compression zone can be expressed as

(12) $$C=\int_{0}^{c_{s}}\sigma (\varepsilon )bdx=\int_{0}^{x_{0}}f_{c}b[1-{\left(1-\frac{\varepsilon }{\varepsilon_{0}}\right)}^{2}]dx+\int_{x_{0}}^{c_{s}}f_{c}bdx=f_{c}bc_{s}(1-\frac{1}{3}\times \frac{\varepsilon_{0}}{\varepsilon_{c}})$$

where *b* is the beam width.

The distance from the resultant force *C* to tip of critical diagonal crack can be estimated as

(13) $$z=\frac{\int_{0}^{c_{s}}\sigma (\varepsilon )bxdx}{C}=\frac{[\frac{1}{2}-\frac{1}{12}{\left(\frac{\varepsilon_{0}}{\varepsilon_{c}}\right)}^{2}]c_{s}}{1-\frac{1}{3}\times \frac{\varepsilon_{0}}{\varepsilon_{c}}}$$

Combining Equations (9) and (13) with $\lambda =\left[\frac{1}{2}-\frac{1}{12}{\left(\frac{\varepsilon_{0}}{\varepsilon_{c}}\right)}^{2}\right]/\left(1-\frac{1}{3}\times \frac{\varepsilon_{0}}{\varepsilon_{c}}\right)$, the height of concrete compression zone above critical diagonal crack is determined by

(14) $$c_{s}=\frac{0.07T_{y}a+T_{\mathrm{ob}}(0.07a\cos\beta +0.13h_{0}\sin\beta )+0.2T_{x}h_{0}}{T_{x}(1-\lambda )+0.5T_{y}a/h_{0}+T_{\mathrm{ob}}(0.5\sin\beta -\lambda \sin\beta +0.5a/h_{0}\cos\beta )}$$

Then, the resultant force *C* and the distance *z* can be determined from Equations (12)–(14). In addition, as Figure 3 shows, the equilibrium of horizontal forces on the free-body diagram above critical diagonal crack is determined by

(15) $$C-T_{x}-T_{\mathrm{ob}}\sin\beta =0$$

Thus, the longitudinal reinforcement force can be further expressed as

(16) $$T_{x}=f_{c}bc_{s}(1-\frac{1}{3}\times \frac{\varepsilon_{0}}{\varepsilon_{c}})-A_{\mathrm{eff},\mathrm{ob}}f_{\mathrm{yc},\mathrm{ob}}\sin\beta$$

As previously noted, the beam shows shear-compression failure when *R* = *V*_u_. From Equations (8), (12)–(14) and (16), the shear capacity of RC member can be expressed as

(17) $$V_{u}=\alpha_{1}A_{\mathrm{eff},y}f_{\mathrm{yc},y}+\alpha_{2}A_{\mathrm{eff},\mathrm{ob}}f_{\mathrm{yc},\mathrm{ob}}+\alpha_{3}f_{c}bc_{s}$$

(18) $$\alpha_{1}=\frac{0.5(h_{0}-c_{s})}{h_{0}}$$

(19) $$\alpha_{2}=\alpha_{1}\cos\beta -\alpha_{1}\frac{h_{0}}{a}\sin\beta$$

(20) $$\alpha_{3}=(1-\frac{1}{3}\times \frac{\varepsilon_{0}}{\varepsilon_{c}})\frac{h_{0}-c_{s}+z}{a}$$

In the next section, the test process incorporating material properties, geometric dimensions, corrosion test, and loading method is provided. The effects of corroded shear steel bars on deflection, failure mode and shear strength are discussed. Shear strength prediction using the proposed model is presented.

## 3. Experimental Program and Model Validation

### 3.1. Experimental Program

**Figure 5:**
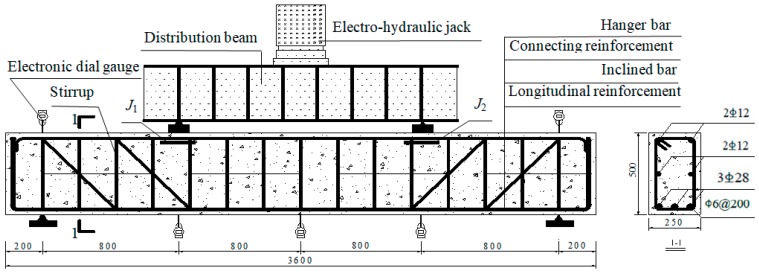
Details of experimental design (unit: mm).

Fourteen members were manufactured for assessing the influence of corrosion in stirrups and inclined bars on shear performance. The beam had a cross section of 250 mm × 500 mm. The length of beam was 3600 mm. Hot rolled reinforcements were used in these specimens. The diameters (d) of longitudinal, hanger and inclined bars were 28, 12 and 12 mm, respectively. All three types of reinforcing bars were HRB335 deformed rebar. The stirrups were double-legged plain bar (HPB235) with 6 mm diameter and 200 mm spacing. Figure 5 shows the dimension and reinforcement of these test specimens. The material properties of reinforcements are shown in Table 1.

An electrochemically accelerated corrosion method was applied to obtain various corrosion losses of rebars. These test specimens were designed as follows: Group A with varying corrosion levels only related to stirrups; and Group B with varying corrosion levels for all steel bars. A-0 and B-0 are uncorroded beams. The specimens were placed in the 5% chloride sodium solution. The steel bars that intended to be corroded were anode. The cathode was additionally provided. An imposed current density was 0.0183 mA/mm^2^. The conduction time was controlled based on Faraday’s law. A difference generally exists between actual corrosion rate and theoretical estimation. The mass loss caused by corrosion was selected to represent the corrosion degree. The reinforcements were removed from the failed members. The corrosion degree was obtained by measuring the mass difference before and after corrosion. More details on the measurement can be found in [3,11,40]. It should be noted that pitting corrosion may have a more serious influence on the shear strength due to the pit geometry and location being random [41], which is beyond the scope of this study. The corrosion levels (average mass loss) for different types of reinforcements are shown in Table 2.

A monotonously static loading test was carried out when the specimen reached the designed corrosion level. Figure 5 shows the loading system. The shear span ratio was 1.75. The deflections at different locations were measured during the entire loading process. The *j*_1_ and *j*_2_ strain gauges (see Figure 5) were arranged at the lower part of the loading point (the upper side of member) for measuring horizontal compressive strain. The propagation of concrete crack was observed, and the width of the cracks were also measured.

### 3.2. Result and Discussion

**Figure 6:**
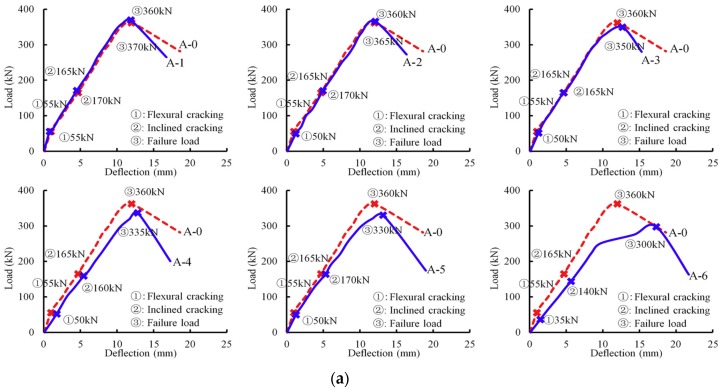

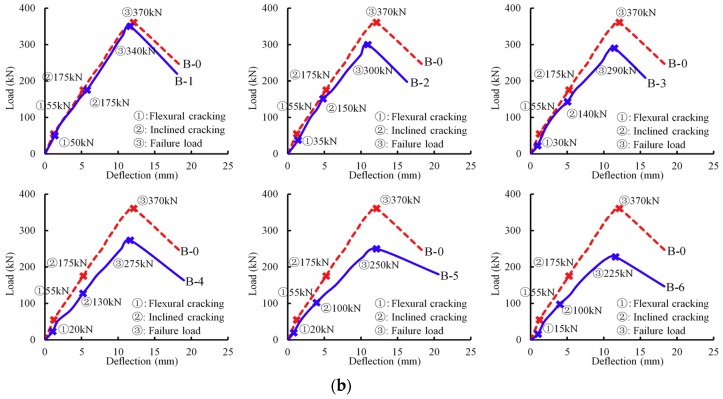
Load–deflection curves at mid-span: (**a**) Group A; and (**b**) Group B.

Figure 6 depicts different load–deflection curves at the mid-span of beams. For beams in Group A, the deflections of the slightly corroded beams A-1 and A-2 at the ultimate load were 2.1% and 0.9% larger than that of the virgin member. The deflections for the severely corroded beams A-3–A-6 at the failed load were 4.5%, 6.5%, 9.1% and 43.6% higher than that of the virgin specimen, respectively. The observations indicate that the stirrup corrosion did not have significant effect on the initial stiffness. However, the severely corroded stirrup had a relatively larger effect on the deterioration of the initial stiffness. For example, beam A-6 had the maximum corrosion level in Group A, and an obvious stiffness reduction was observed. Figure 6b is the mid-span deflections of beams in Group B. A similar phenomenon that the corroded beams exhibited a reduction in deformation was also observed. For members B-2–B-6, the slopes of these curves were less than that of the virgin beam.

**Figure 7:**
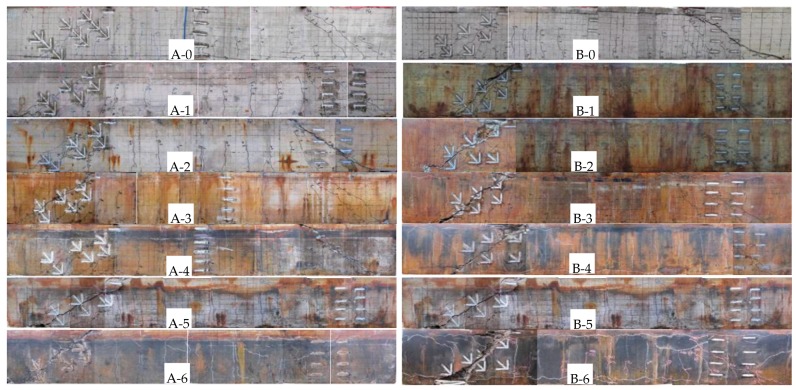
Failure modes for experimental members.

The applied loads corresponding to the occurrence of different types of cracks were also recorded. Figure 6 presents the loads corresponding to flexural cracking, inclined cracking and failure. As can be seen, the load data of the control beam are shown above the curves; the load data of these deteriorated members are listed blow the curves. The diagonal cracking strength of beams in Group B decreased significantly. One of these diagonal cracks extended to the loading point and gradually developed into the critical diagonal crack. These crack morphologies of test members are shown in Figure 7. The test specimens in the two groups presented the same failure mode. Due to the corrosion in different steel bars, the two groups exhibited little difference in the deterioration process. Specimens A-4–A-6 with severe corrosion showed the fracture of stirrups. The corrosion level in Group B was larger than that of Group A, and fracture of both the stirrups and inclined reinforcements were found. In addition, the reinforcement corrosion in Group B led to a change of the inclination angle of the critical diagonal crack, which differed from Group A. For beam B-0 and corroded beams B-1–B-3, the inclination angle of critical diagonal crack was small, which propagated to a loading location. The severely corroded beams B-4–B-6 had a larger inclination angle of the critical diagonal crack.

**Figure 8:**
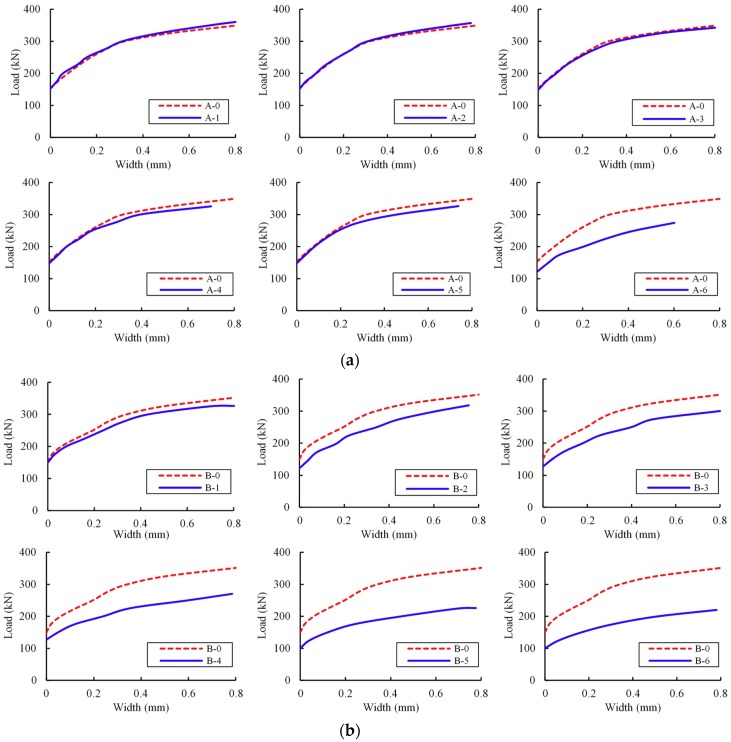
Load–maximum diagonal crack width curves: (**a**) Group A; and (**b**) Group B.

Figure 8 shows the maximum diagonal crack width under various loads. From load at which crack initiates to the ultimate load, a nonlinear increase of diagonal crack width was observed as load increased. For the slightly corroded specimens in Group A, the diagonal crack width propagation was similar for beams A-0, A-2, and A-3. The moderately corroded beams A-4 and A-5 exhibited slightly quicker crack propagation. The severely corroded beam A-6 experienced an obvious deterioration due to concrete section damage. The observation indicates that slight stirrup corrosion had a negligible influence on diagonal crack propagation. The crack propagated quickly with the appearance of the corrosion-caused cracks. Figure 8b depicts different diagonal crack propagation curves for beams in Group B. All critical crack propagation curves exhibited remarkable distinction in different specimens, which differed from that of Group A. The corrosion in all shear steel bars accelerated diagonal crack propagation.

The above observations indicate that the increased corrosion level accelerated the growth of diagonal crack width. As corrosion propagated, the restraining effect of stirrups and inclined steel bars on the concrete deteriorated. In addition, the increase of the horizontal corrosion-induced crack width along the longitudinal steel bar accelerated the propagation of the diagonal crack.

### 3.3. Model Validation

**Figure 9:**
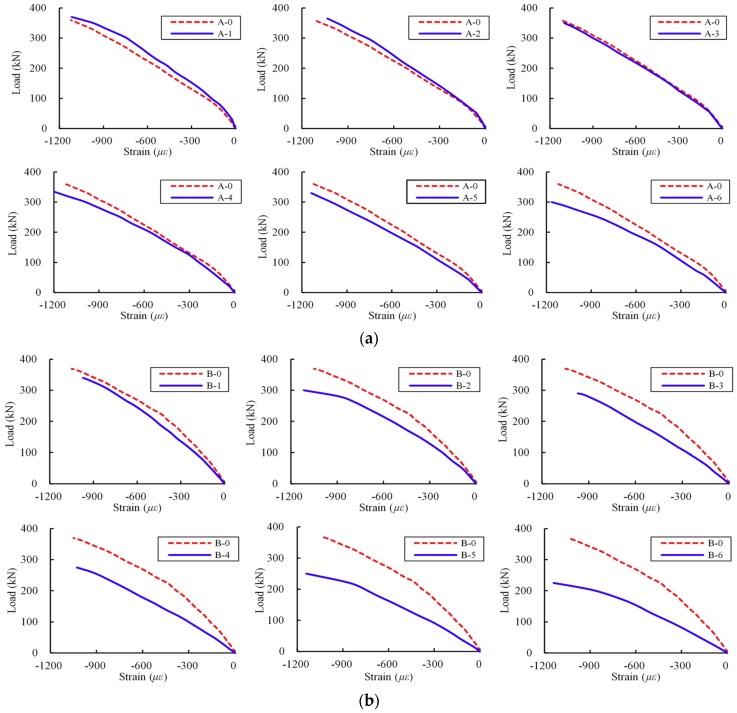
Load–strain curves in shear-bending section of concrete: (**a**) Group A; and (**b**) Group B.

As previously mentioned, a key parameter for the shear strength prediction model is the concrete compressive strain in the shear compression zone. Figure 9 shows the load–strain curve of concrete in the shear-bending zone at the tip of critical diagonal crack. As Figure 9a shows, at a load of 200 kN, the horizontal compressive strains of slightly corroded beams A-1 and A-2 were 16.1% and 10.1% smaller than that of beam A-0, respectively. The stains of corroded beams A-3–A-6 were 2.1%, 7.8%, 18.2% and 21.4% larger than that of beam A-0, respectively. As Figure 9b shows, the horizontal compressive strains for deteriorated members B-1–B-6 were 17.3%, 37.1%, 57.0%, 79.9%, 90.7% and 121.4% larger than that of beam B-0, respectively.

For test beam with low corrosion level, the increased volume caused by corrosion product increased the friction between the reinforcement and concrete, which resulted in a certain increase in the bond strength and enhanced the synergistic performance between reinforcement and concrete. Therefore, the horizontal pressure resistance of the concrete in the shear zone increased. As corrosion increased, the height of the compressive concrete at the tip of critical diagonal crack decreased. Then, the horizontal compressive strains of the concrete gradually increased. The concrete section damaged in the shear-bending section weakened the shear capacity of concrete members. The compressive edge strains of concrete are listed in Table 2.

**Figure 10:**
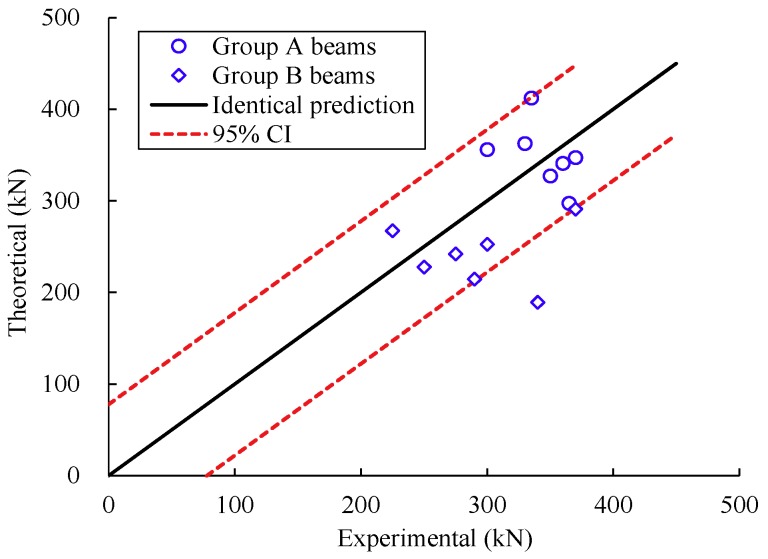
Comparison between calculated and experimental results.

Figure 10 shows a comparison between the predictions and the test observations. *x*-axis and *y*-axis represent the experimental results and the theoretical results, respectively. A 95% confidence interval (CI) of the predicted value is also given (see the dashed lines). As can be seen, most of the theoretical values were close to the test observations and 92.9% of the data fell within this confidence interval. The mean of the ratio of the predicted values to the observation value was 0.93. The standard deviation was 0.18. Few test points were outside the 95% confidence bounds. The points with large deviations were mainly for the beams with small strain in the concrete compression zone, especially for beams B-1 and B-3, which may be attributed to the effects of test environment and strain gauge on the measurement of the concrete strain.

## 4. Conclusions and Further Study

An experimental work was conducted to evaluate the shear strength deterioration of corroded RC members. Load–deflection, critical diagonal crack propagation and failure modes were considered. The experimental observations indicate that the corrosion of different types of reinforcements had little effect on the failure mode of the test members under a determined shear-to-span ratio. A shear strength model for the members with inclined rebars was established based on a limit equilibrium method. The theoretical prediction model is mainly composed of the tensile strength of longitudinal steel bars, the shear force provided by the concrete on the top of diagonal crack and the tensile force of the shear steel at the diagonal crack. The degradation of the cross-sectional area and strength of reinforcements caused by corrosion are also considered. It was found that the height of compression zone above critical diagonal crack was one of the most influential factors for the shear capacity of RC beam. This proposed method can be applied for both corroded and uncorroded reinforced concrete members.

The corrosion levels obtained from the accelerated corrosion test may differ from the natural environment. The application of the findings in this study to other rear engineering condition may be limited. The shear capacity of corroded RC members is also related to shear span-to-depth ratio, loading conditions, reinforcement ratio and other possible influential factors. The proposed prediction method needs further validation due to limited sample size. Future theoretical and experimental studies are also required to include additional source uncertainty for a probabilistic prediction model.

## Figures and Tables

**Table 1:** Details of various reinforcements.

Grade	Steel Bar	d/mm	$f_{y0}/\mathrm{MPa}$	Maximum Strength/MPa
HRB335	Longitudinal bar	28	365.70	519.30
HRB335	Hanger bar	12	369.30	513.60
HRB335	Inclined bar	12	367.10	517.10
HPB235	Stirrup	6	337.90	482.20

**Table 2:** Summary of experimental results.

Beams Numbers	$\eta_{x}/\%$	$\eta_{ob}/\%$	$\eta_{y}/\%$	*f*_c_/MPa	$\varepsilon_{c}$ /με
A-0	0.00	0.00	0.00	36.5	−1121
A-1	0.00	0.00	4.23	37.5	−1116
A-2	0.00	0.00	6.25	36.5	−1034
A-3	0.00	0.00	11.23	35.4	−1102
A-4	0.00	0.00	21.57	38.3	−1248
A-5	0.00	0.00	22.51	37.2	−1138
A-6	0.00	0.00	36.33	37.5	−1165
B-0	0.00	0.00	0.00	34.8	−1050
B-1	4.27	12.07	18.11	27.9	−972
B-2	6.32	14.54	29.17	28.7	−1116
B-3	6.73	14.62	31.14	32.6	−970
B-4	8.41	20.73	43.51	31.9	−1027
B-5	9.52	28.42	53.79	26.0	−1141
B-6	11.42	34.96	68.35	31.8	−1139

$\eta_{x}$, $\eta_{ob}$ and $\eta_{y}$ are the corrosion levels of longitudinal steel bar, diagonal reinforcement and stirrups, respectively.
